# Update on Reovirus Infections in Waterfowls

**DOI:** 10.3390/ani15203053

**Published:** 2025-10-21

**Authors:** Szilvia L. Farkas, Zsófia Lanszki, Yashpal S. Malik, Vito Martella, Vilmos Palya, Krisztián Bányai

**Affiliations:** 1 Department of Obstetrics and Food Animal Medicine Clinic, University of Veterinary Medicine, 1078 Budapest, Hungary; fszilvi@yahoo.com; 2 National Laboratory of Virology, Szentágothai Research Centre, University of Pécs, 7624 Pécs, Hungary; lanszkizsofi@gmail.com; 3 Institute of Biology, Faculty of Sciences, University of Pécs, 7624 Pécs, Hungary; 4 ICAR-Indian Veterinary Research Institute (ICAR-IVRI) Mukteswar, Nainital 263138, India; malikyps@gmail.com; 5 Department of Veterinary Medicine, University of Bari Aldo Moro, 70010 Valenzano, Italy; vito.martella@uniba.it; 6 Department of Pharmacology and Toxicology, University of Veterinary Medicine, 1078 Budapest, Hungary; 7 Ceva-Phylaxia Ltd., 1107 Budapest, Hungary; vilmos.palya@gmail.com; 8 Department of Medical Biology, Medical School, University of Pécs, 7624 Pécs, Hungary; 9 National Laboratory of Infectious Animal Diseases, Antimicrobial Resistance, Veterinary Public Health and Food Chain Safety, HUN-REN Veterinary Medical Research Institute, 1143 Budapest, Hungary

**Keywords:** duck, goose, *Orthoreovirus*, disease monitoring, genome sequencing, genotyping, vaccine development

## Abstract

**Simple Summary:**

Reoviruses cause a range of diseases in a number of aquatic wild birds and domestic waterfowl species, including Pekin ducks, Muscovy ducks and geese, impacting poultry health and production worldwide. This review provides an update on reovirus infections in waterfowl, with a particular emphasis on recent advances concerning their surveillance and control.

**Abstract:**

Reovirus infections pose a significant threat to waterfowl health and productivity globally. This review provides a comprehensive update on various aspects of waterfowl reoviruses (WRVs) affecting domestic duck and goose species. We outline the genetic diversity and evolution of circulating strains. The paper details the array of clinical signs and pathologies observed in infected birds. Most advanced laboratory diagnostic methods, including molecular techniques, are reviewed for their role in rapid and accurate detection, forming the cornerstone of effective surveillance programs. Furthermore, we explore the advancements in WRV vaccine development, covering traditional as well as promising novel approaches. The ongoing challenge of managing WRV infections necessitates integrated surveillance-control programs, prioritizing enhanced diagnostic capabilities and the development of more efficacious and broadly protective vaccines to safeguard populations of domestic ducks and geese.

## 1. Introduction

Waterfowl farming is a major segment of global animal agriculture. This sector continues to grow owing to the strong demand, especially in Asian markets [1]. Despite the trends of growing waterfowl production, intensive farming faces significant challenges from infectious diseases. These diseases cause substantial economic losses through high morbidity and mortality, reduced productivity, increased treatment costs, and trade restrictions. Most challenging viral diseases of waterfowl include duck plague, parvovirus infection, viral hepatitis, and avian influenza, each of which is associated with mortality rates above 50% [2,3,4,5].

Reoviruses are responsible for various illnesses in ducks and geese [2]. Disease severity and economic impact depend on several factors, including the virus strain, host species and breed, as well as bird age and the presence or absence of maternally derived antibodies. While some reovirus disease outbreaks are characterized by as high as 50% mortality, most field reports show significantly lower rates [2,6,7,8,9]. Reovirus infections in waterfowl have been known for seven decades [10], yet the recent emergence of a novel, more lethal variant of waterfowl reovirus (WRV) around the millennium sparked renewed interest, leading to better surveillance based on new diagnostic methods and the development of new vaccine candidates.

This narrative review addresses the current landscape of waterfowl reovirus infections in light of renewed scientific interest. We provide an overview of key areas, including clinical disease and pathology, new viral diversity, improved disease surveillance tools, and contemporary vaccine development strategies.

## 2. Classification, Genomic Features

Reovirus-related disease was first documented in Muscovy ducks in South Africa in the 1950s [10]. The first report of reovirus disease in geese originated from Hungary in the 2000s [8]. Starting from the mid-2000s, a new reovirus disease emerged in China in different waterfowl species and the isolated viruses were genetically distinct from the European-origin WRVs [11,12,13,14]. This led to the introduction of the terms “classical WRVs” (also called MDRV for Muscovy duck reovirus and GRV for goose reovirus) and “novel WRVs” (originally NDRV or novel duck reovirus). So far, disease outbreaks caused by novel WRVs have been reported exclusively from Asia. In the 2010s, a genetically distantly related group of WRVs has been identified, with only a few strains reported from China and Europe [15,16,17]. Additionally, evidence from field reports and infection experiments indicate that waterfowls are susceptible to infection by reoviruses typically found in chickens [18,19].

**Figure 1:**
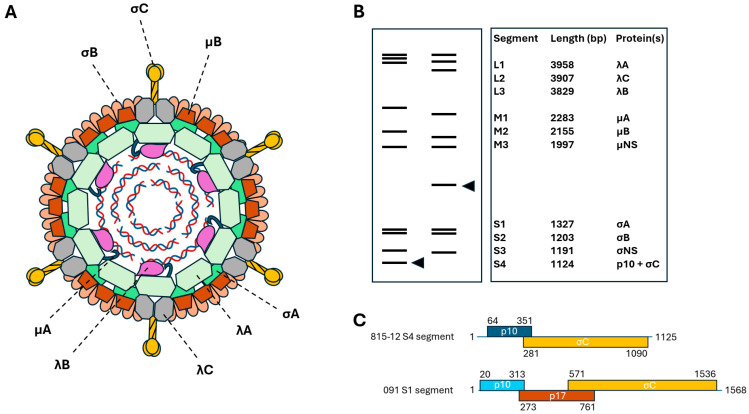
Some structural characteristics of waterfowl reovirus particles and genomes. Panel (**A**). Schematic structure of reovirus virion. Core proteins: λA, λB, λC, μA, and σA; outer capsid proteins: μB, σB, and σC. Panel (**B**). Schematic RNA profile of classical WRV (RNA profile on the left) and novel WRV (RNA profile on the right) with the relative position of the σC coding genome segment (arrow head), and the length of segments and the encoded proteins on the right. Panel (**C**). Open reading frames and encoded proteins of the S1/S4 segment in a classical (815-12; GenBank acc.no., KC508656) and a novel (091; GenBank acc.no., JX478256) WRV strain.

Both classical and novel WRVs belong to the *Avian orthoreovirus* species (genus *Orthoreovirus*, family *Spinareoviridae*, order *Reovirales*, class *Resentoviricetes*, phylum *Duplornaviricota*) [20]. The virus particles are non-enveloped with a size range of 70–80 nm. The viral genome is a 10-segmented double-stranded RNA, and the combined length of the RNA segments is about 22.9 to 23.4 kilobase pairs [21,22]. The coding regions are flanked by untranslated regions at both the 5′ and 3′ ends, whose lengths fall between 11 nt and 62 nt and 32 nt to 101 nt, respectively, depending on virus strain [21,22]. The genome segments are categorized by size into three large (L), three medium (M), and four small (S) segments. Apart from the S1 or S4 segments, which are bi- or tri-cistronic (depending on viral lineage), each segment encodes a single protein (mono-cistronic). Among the 11 or 12 primary translation products, eight are involved in the formation of the multi-layered virion (Figure 1A), with five proteins within the core (λA, λB, λC, μA, and σA) and three proteins within the outer layer (μB, σC, and σB) [23,24]. The remaining products are non-structural proteins (μNS, σNS, p17, and/or p10) that play basic roles in virus multiplication, such as viroplasm formation, modulation of cellular processes to favor viral replication, and facilitating cell fusion [23,24]. Of note is that the fusogenic capacity is not a universal characteristic among viral lineages of WRVs [17,21,25,26,27,28].

When using RNA polyacrylamide gel electrophoresis, a technique that separates viral genome segments by size, classical and novel WRVs show differences in how their polycistronic S1/S4 segments migrate [29,30]. In polyacrylamide gels, these genome segments move faster in classical WRVs than in novel WRVs (Figure 1B), a finding that is consistent with structural differences of the corresponding genome segments. In fact, in novel WRVs, the largest S-class segment (S1) is structurally similar to chicken and turkey origin reoviruses. The S1 segment contains genes for the cell-attachment protein, σC, and two non-structural proteins, p10 and p17 (also referred to as p11 and p18, respectively, in other sources), in a partially overlapping arrangement [23,24] (Figure 1C). The p17 protein is involved in modulating host cell responses to infection through its interference with specific nuclear and cytoplasmic processes. Conversely, the p10 protein, which belongs to the FAST (fusion-associated small transmembrane) protein family, mediates syncytium formation in cell culture, a feature observed in numerous, but not all, WRVs [30,31]. Notably, structural alterations in the multimerization sequence motif of p10 can abolish the fusogenic capacity in certain novel WRVs [30,31]. In classical WRVs, the S4 segment is bi-cistronic and encodes the σC and p10 proteins [32,33,34] (Figure 1C). However, the p10 protein in classical WRVs is not an ortholog of the p10 protein found in the fusogenic novel WRVs and cannot consequently form syncytia. Instead, experiments have shown that the expression of p10 protein of classical WRV leads to cell cycle arrest and apoptosis [35].

**Figure 2:**
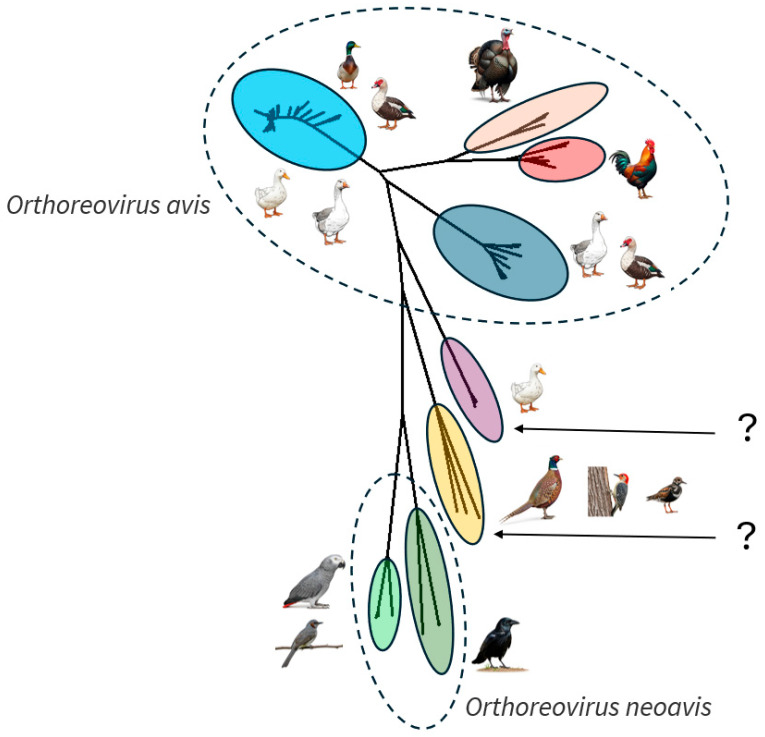
Unrooted neighbor joining tree of the σB protein. Avian-origin orthoreoviruses are currently classified into two established species, *Orthoreovirus avis* and *Orthoreovirus neoavis*. Question marks indicate newly described genetic clusters whose taxonomic position is unclear and might represent putative new orthoreovirus species. The cluster highlighted in purple contains the third lineage of WRVs isolated from Pekin ducks in Germany, China, and Hungary.

Newly identified WRVs, which do not fit into existing WRV pathotypes or genotypes, have a tri-cistronic S1 segment but show much lower genetic similarity to other ARVs across their entire genome (gene-wise ranges from 36% to 72% for classical WRVs, from 39% to 72% for novel WRVs, from 33% to 72% for chicken origin reoviruses [15,16]. The sequence homology of these distantly related WRVs is puzzling and current classification criteria do not allow them to be firmly classified within the *Orthoreovirus avis* or the *Orthoreovirus neoavis* species or to establish a new species (Figure 2). The International Committee on Taxonomy of Viruses uses a nucleic acid similarity below 60% to separate reovirus species, while a similarity above 75% indicates the same species [20]. This leaves a wide “grey zone” between these thresholds. For all major genes, this third genetic lineage of WRVs is characterized by similarity values falling within or below the grey zone [15,16]. Accordingly, their precise classification requires formal confirmation by virus taxonomists.

## 3. Epidemiology, Evolutionary Mechanisms

WRVs typically cause disease in young birds, but waterfowl of all ages can be susceptible to a productive virus infection [8,26]. Transmission occurs through the fecal-oral route, via airborne droplets, or parenterally, often through the skin of the footpad. Infection experiments confirm the susceptibility of hosts through different routes of infection [17,26,27]. More recently, evidence of vertical transmission has also been reported [36].

Classical WRVs have been reported to occur in African, European, and Asian countries and are known to cause pathology in Muscovy ducks and domestic geese [7,8,10,37,38,39,40]. Novel WRVs are known to occur in Asia. The host range for these novel WRVs is broader, with the disease and pathogen detected in Muscovy ducks, Pekin ducks, mallards (*Anas platyrhynchos*), swans, and geese [9,11,14,41,42,43]. Concerning the third genetic lineage of WRVs, which is distinct from the classical and novel WRVs, only three independent isolates have been characterized in detail—one each in Germany, Hungary, and China—all from domestic Pekin ducks [15,16,17]. In these cases, the clinical symptoms and pathological findings were reported to be mild and atypical, raising speculations concerning their etiologic role. A reovirus with an undetermined genetic background was isolated in Finland from a highly fatal outbreak in young common eider (*Somateria mollissima*), where only 1–5% of the chicks survived. The affected chicks were up to 3 weeks old. Interestingly, experimental infection of mallards with this eider-derived reovirus did not result in mortality [44]. Researchers from Australia have recently deposited WRV sequences from Pacific black duck (*Anas superciliosa*) as part of virus surveillance using viral metagenomics, providing evidence for WRV circulation in Australia and for additional viral diversity [45].

Typically, WRVs can be differentiated from chicken- and turkey-origin reoviruses by sequencing and phylogenomic analyses, although some genes of different WRV lineages tend to show genetic relatedness with heterologous reovirus strains [21,28,34]. These somewhat controversial features can be derived from two primary evolutionary mechanisms, such as point mutations leading to genetic drift and reassortment of homologous gene segments, leading to genetic/antigenic shift.

The accumulation of point mutations is driven by the lack of a proofreading mechanism in the viral replication machinery and plays a fundamental role in genetic variability. The mean rate of evolution of avian-origin reoviruses was estimated 2.3 × 10^−3^ substitution/site/year [46]. Circulating WRV strains maintain high nucleotide sequence identity (≥85%) among themselves, although phylogenetically, their genes tend to segregate into distinct genotypes. Furthermore, these WRVs are genetically distant from chicken and turkey reoviruses, sharing less than 80% nucleotide sequence identity [21,28,34,47,48].

**Figure 3:**
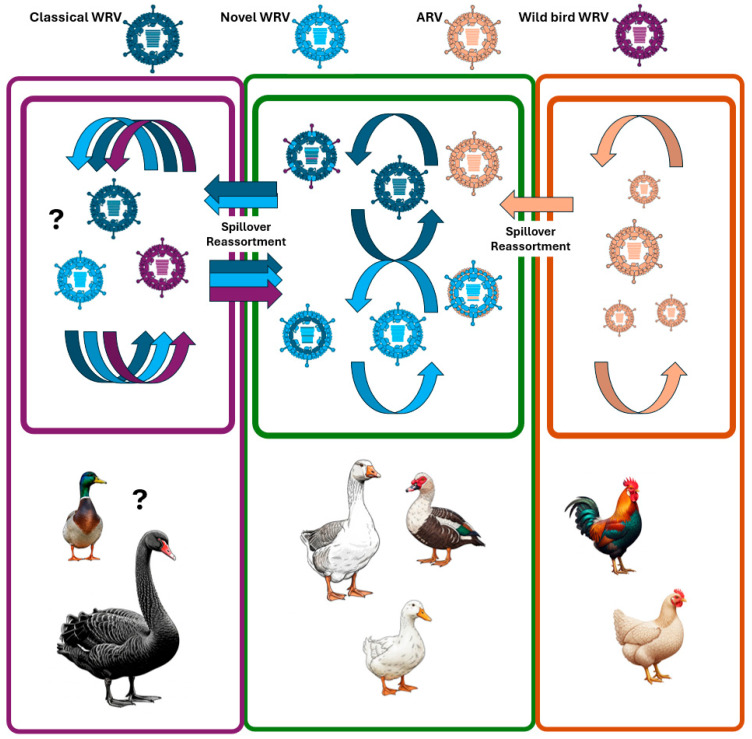
Reoviruses exhibit significant diversity in domestic ducks and geese, with evidence pointing to the circulation of two major pathotypes (represented by light and dark blue particles and arrows). These viruses predominantly circulate within their respective geographic areas, undergoing extensive genetic reassortment involving nearly their entire set of genome segments. Such reassortants are known to occur in both Europe and Asia. Further reovirus strain diversity exists within wild Anseriform birds. Reoviral strains from these wild birds can directly transmit to domestic waterfowl or contribute to novel allele constellations through reassortment. While avian reoviruses of chicken origin typically circulate within chicken farms, these strains can occasionally infect domestic waterfowl and upon infection they may donate genome segments, leading to the creation of novel reassortants.

This picture is further complicated and the diversity of WRVs increases by the generation of new allele combinations resulting from reassortment of cognate genome segments. Numerous genes seem to be involved in reassortment events between classical and novel WRVs [34]. In addition, viable progeny viruses have been described, with some reoviral alleles originating from heterologous hosts (Figure 3). An intriguing example of a recent reassortment event was the identification of a mono-reassortant duck isolate with an acquired S3 genome segment and a double-reassortant goose isolate with acquired S3 and S4 genome segments, one each obtained from chicken-origin reovirus strains [49]. Another strain from Germany, which harbored a mixture of classical and novel WRV genes, was found to possess a *σC*-coding gene with limited sequence homology (nt, ~76%; aa, ~83%) to those of novel WRVs, thereby raising questions about the host species origin of its reassorted S1 genome segment [15]. Given that reassortment occurs when different viruses infect the same cell, the direct interspecies transmission of a heterologous reovirus to waterfowl is a prerequisite for the subsequent selection of any mono- or multi-reassortant WRV progenies.

Recent data have also indicated that recombination can occur among the homologous genes of WRVs [48,50]. Furthermore, the structural distinction in the *σC*-coding S-class genome segment, differentiating tri-cistronic novel WRVs from bi-cistronic classical WRVs, could have arisen from deleterious mutations within the p10/p17 region of the S1 genome segment or through independent recombination with heterologous RNA(s) [15,31]. Additionally, deleterious mutations affecting an 18-amino-acid-long fragment in the *σC* coding gene have been seen in a circulating strain with common genetic composition that was reported to display increased virulence [51].

A fundamental question in the field is how specific constellations of viral genes determine virulence and pathotypes. Although the amount of whole-genome sequence data is expanding, it remains insufficient to provide a conclusive answer. Nevertheless, a noteworthy observation emerged from early phylogenetic studies. While reassortment between novel and classical WRVs may affect most of the genome, a significant genetic linkage may exist among the segments encoding the μB, σB, and σC proteins. Moreover, the specific constellation of these genes appeared to co-segregate with the observed pathotypes of WRV strains [34]. In this context, the role of other genes or specific mutations within any genome segments remains a significant knowledge gap, which appears to be worth exploring now that a reverse genetics platform is available for WRVs [52].

## 4. Clinical Signs, Pathology

Disease associated with reovirus in Muscovy ducks was first reported in South Africa [10]. Ducks of all age groups were affected, but mortality was highest in 8-to-10-week-old ducks. Infected birds displayed fever, general weakness, anorexia, diarrhea, and constant thirst. Birds surviving the acute phase of the disease often developed locomotor disorders and chronic lameness. Similar clinical signs were observed in subsequent disease outbreaks in Muscovy ducks in France and Israel, from which the virus was isolated [7,37].

With classical WRV infections, mortality is relatively low, usually under 20%, whereas novel WRV infections can lead to much higher mortality, up to 50% [2,6,7,8,9]. Experimental infections with classical WRVs in Muscovy ducks have successfully reproduced the disease, though often in a milder form and with lower mortality than seen in field outbreaks. In contrast, infection experiments with novel WRVs consistently resulted in severe symptoms and significant mortality [6,14,51,53].

Reovirus infection in young domestic geese has been predominantly observed in meat-type geese, between 2 and 8 weeks of age [8]. The clinical signs in the acute phase of the disease include general weakness, diarrhea, and reduced mobility. Survivors of the acute phase may continue to show locomotor disorders and lameness. Mortality in classical WRV infections in geese is moderate (2–10%), primarily among young birds, while older animals might only show locomotive disorders, such as the swelling of hock, metatarsal, digital joints, and synovial bursae, frequently resulting in the rupture of the flexor tendon. In contrast, novel WRV infections may cause mortality rates as high as 40% in geese, and the pathological presentation is also more severe [8,19].

The pathology of WRV infections has been well documented. For example, in ducks, WRV infections cause characteristic lesions in the internal organs, which are milder in the case of classical WRV infections and more severe with novel WRV infections. The liver and spleen are most frequently affected, appearing moderately enlarged with multiple greyish-white, pinhead-sized foci. Hemorrhages under the epicardium and pericarditis are common. The kidneys are also enlarged and show hemorrhages. The spleen and not consistently the bursa show atrophy in chronic cases [6,7,9,10].

Experimental Muscovy duck infections have confirmed these characteristic pathological lesions, including the enlargement of liver and spleen (hepato-splenomegaly) with multiple inflammatory necrotic foci. Histopathological examination has revealed extensive hepatocyte degeneration and necrosis, accompanied by inflammatory cell infiltration. The inflammatory necrotic foci in the liver and spleen typically consisted of a necrotic center with epithelial cells, fibroblasts, and multinucleated giant cells at the periphery. Focal hepatic necrosis and proliferation of bile ducts were also observed [6,7,9,10]. Another hallmark of the disease is inflammation of leg joints and their capsules, with lymphocytic-plasmacytic infiltration around the joint tissues. Alongside these inflammatory and necrotic reactions, lymphocyte depletion was apparent in the medullary areas of bursa Fabricius follicles [14,54].

**Figure 4:**
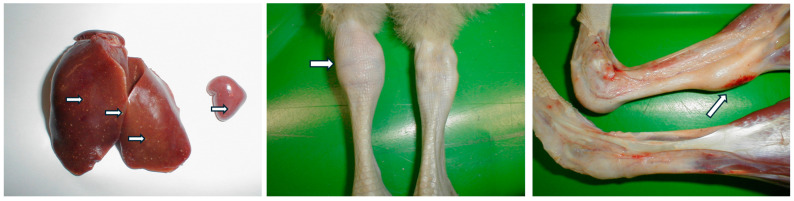
Yellowish-grey necrotic foci caused by reovirus infection in the liver and the spleen of gosling (**left**). Arthritis and tenosynovitis in gosling (**middle**) and hemorrhages in the leg caused by the rupture of the gastrocnemius tendon (**right**). (Photograph by the author, V. Palya). Arrows highlight the respective pathologies.

In geese, reovirus infection shows a similar pathological presentation to that seen in ducks. The liver and spleen are moderately enlarged, often with multiple grayish-white foci (Figure 4). Histologically, miliary foci of vacuolated, necrotic hepatocytes and granuloma-like foci with necrotic centers and proliferating macrophages and epithelial cells at the periphery were seen in the liver. In the periportal region, infiltration by heterophil cells and histiocytes, and proliferation of bile ducts was visible. The spleen exhibited similar changes, with a significant reduction in lymphoid follicles. In chronic cases, the acute phase lesions observed in the liver and spleen could not be seen. The kidneys were swollen with hemorrhages, and some degeneration of the tubular epithelium. Sero-fibrinous pericarditis, infiltration of the subepicardial region by mononuclear cells could also be seen [8,19].

The development of sero-fibrinous inflammation of hock, metatarsal, or digital joints, as well as the gastrocnemius and digital flexor tendons, tendon sheaths and bursas during the subacute and chronic phase of the disease is characteristic in domestic geese (Figure 4). Besides the arthritis and tenosynovitis, sero-fibrinous inflammation of the synovial bursae is also frequently seen. The synovial layers of the tendon sheaths are infiltrated with different mononuclear inflammatory cells, and organization of the fibrinous exudate occurs. In chronic cases, connective tissue granulating from the subsynovial layers of tendon sheaths accumulates, which sometimes invades the tendon, and the two membranes of the tendon sheaths frequently become adhered. As a consequence, rupture of the damaged tendons can occur, leading to tissue hemorrhages, especially in the gastrocnemius tendon area (Figure 4). Similarly to ducks, in the bursa of Fabricius lymphocyte depletion and sometimes necrosis can be seen in the medulla of the follicles. Less common findings include air-sacculitis and enteritis [8,19].

## 5. Laboratory Diagnosis

An accurate diagnosis of reovirus infection in waterfowl is complicated by nonspecific clinical signs, such as inflammatory joint swelling, lethargy, and anorexia. At post-mortem examination, characteristic lesions in the liver and spleen, pericarditis, and significant involvement of the metatarsal and toes joints and flexor tendons can be suggestive of reovirus infection. The establishment of a definitive diagnosis, however, should be supported by the direct detection of the virus from the affected tissues [55].

Historically, the diagnosis of waterfowl reovirus (WRV) infection depended on clinical signs, pathological lesions, and virus isolation [7,8,10,37]. These conventional techniques are often too slow for effective routine diagnostics. Virus isolation on cell cultures or embryonated eggs can be performed from tissue homogenates, typically sourced from the liver, spleen, tendon tissue, or joint exudate. The cytopathic effect observed in these cultures can differ among and within WRV genotypes [7,8,30,56]. Immunodiffusion and enzyme-linked immunosorbent assay (ELISA) can be used for serological survey of flocks for detection of infection with any types of WRVs, while indirect immunofluorescence test or virus neutralization have been employed for detection of antibodies against specific strain of WRV, or, for determining antigenic relationship of an isolated strain to reference WRV strains. Of interest, antibodies against the S1133 vaccine strain of chicken reovirus can be used in immunodiffusion and ELISA due to shared antigens among avian reoviruses [57,58,59,60].

In recent years, rapid molecular virological methods have become the preferred tools for WRV diagnostics. Reverse transcription polymerase chain reaction (RT-PCR) is a widely used method for the routine detection of WRV from various tissue homogenates. These assays are designed to target different viral genes depending on the reovirus variant. Common targets include S-class genome segments [8,61]. The past decade has brought continuous advancements in assay formats, leading to the development of conventional multiplex [62,63] and quantitative RT-PCR assays [64,65] (Table 1). More advanced platforms, such as a GeXP analyzer-based multiplex RT-PCR assay, now allow for the simultaneous detection of eleven duck viruses, including classical WRVs [66]. Additionally, a visual gene chip can detect seven waterfowl pathogens at once, including novel WRVs [67], and a one-step multiplex real-time fluorescent quantitative RT-PCR is available for the concurrent detection of Duck Tembusu virus (DTMUV), Duck hepatitis virus (DHV), classical WRV, and Muscovy duck parvovirus (MDPV) [68]. An RT-qPCR assay coupled with high-resolution melting analysis for the simultaneous detection and differentiation of Muscovy duck origin and goose origin reovirus has been developed [69]. These molecular assays exhibit high analytical sensitivity, with detection limits reported as low as 1 copy/µL [63,68], and excellent specificity, with numerous studies confirming no cross-reactivity against a wide panel of other waterfowl pathogens.

Advanced molecular techniques based on isothermal amplification are particularly suitable for field conditions and point-of-care testing, which greatly facilitates epidemiologic surveillance. Notable examples include an RT-LAMP (Reverse transcription loop-mediated isothermal amplification) assay, a method used for the rapid and visual detection of novel WRV, which is highly effective in resource-limited laboratory settings [70]. Another assay is an RT-RPA (Reverse transcription recombinase polymerase amplification), a rapid and visual method developed for the on-site detection of novel WRV [71]. Additionally, a highly efficient molecular detection system for novel WRVs based on CRISPR-Cas13a has been developed for point-of-care testing [72].

Viral metagenomics is a valuable, though less sensitive, method for amplifying WRV genomic RNA from pathological specimens or cell cultures. While its lower sensitivity may preclude it from being a primary diagnostic tool, it excels at identifying fragmented WRV-specific sequence reads. This capability allows for the accurate identification of WRVs [16,21,45], with the potential detection of novel sequence variants that may not be detected by sequence-dependent assays. Furthermore, by enabling the determination of the complete genome sequence of tissue-adapted viruses, this approach is a powerful tool for studying the genetic variability of circulating viruses, making it highly beneficial for molecular epidemiology investigations [15,34].

Ultimately, a comprehensive approach that utilizes multiple diagnostic strategies—combining virus isolation with both classical and advanced molecular detection methods—is essential for the thorough characterization and surveillance of waterfowl reoviruses.

## 6. Disease Control and Prevention, Vaccine Development

Ducks and geese are often raised in semi-intensive housing systems with outdoor runs and swimming areas, which allows for contact with wild birds and increases the risk of pathogen transmission. Since WRVs are ubiquitous in these commercial production environments and in natural ecosystems, keeping flocks entirely free from infection is hard, if not impossible. In the absence of a specific commercial vaccine, prevention relies on rigorous biosecurity and sanitation measures to delay infection and reduce its prevalence. These measures include maintaining high standards of husbandry and hygiene, minimizing exposure to contaminated feed and water, and avoiding multi-age farms where reovirus can circulate between older and younger birds [55].

Given the lack of commercially available vaccines, there is an urgent need for the development of safe and effective alternatives, particularly in Asian countries. While several preclinical vaccine strategies have been explored [73,74,75,76,77,78,79,80], developing an effective vaccine is complicated by the capacity of WRVs to rapidly evolve by mutations, reassortment, and recombination, generating extensive genetic diversity and triggering the emergence of novel variant strains.

Historically, vaccine development efforts often focused on traditional inactivated whole-virus and adjuvanted vaccines [73,74]. While these vaccines can be effective, a major limitation has been described for WRV. Monovalent inactivated vaccines, which contain a single virus strain, provide excellent (100%) protection against challenge with a homologous virus. However, their efficacy drops dramatically against different (heterologous) strains, with protection rates falling as low as 13.3% to 26.7% [74] (Table 2). This poor cross-protection underscores that this traditional approach is insufficient to minimize the impact of the diverse WRV serotypes circulating in the field.

Thus, to achieve more robust protection, the use of a single inactivated virus may not be sufficient. To circumvent this issue, more advanced vaccines have targeted key viral components or addressed serotype diversity directly. Some modern vaccine platforms have been evaluated for WRVs. For example, subunit vaccines use specific purified proteins of the virus instead of the whole virion [75,76,77]. Most recent research has focused on the outer capsid proteins σB and σC, which are major viral antigens known to induce a protective immune response. The σC protein appears to be highly immunoreactive. These proteins have been produced safely in expression systems like the insect cell-baculovirus expression system or bacterial cultures [75,76,77]. Efficacy tests were shown to be promising; a single dose of baculovirus-expressed σC antigen alone or in combination with σB, induced a stronger antibody response than a traditional inactivated whole-virus vaccine. The combined rBac-σB+σC vaccine demonstrated the best overall protective effects, showing superior weight gain and the lowest rate of pathological lesions in the liver and spleen following a viral challenge [77]. Similarly, a subunit vaccine based on recombinant multiple linear B cell epitopes of the σB protein, produced in *E. coli*, provided 100% protection against infection in Muscovy ducks with no clinical signs or lesions observed in challenge experiments [78]. These results collectively show that subunit vaccines are not only effective but can be superior to inactivated whole-virus approaches.

Beyond subunit vaccine platforms, other next-generation strategies have also been evaluated for WRVs. A suicidal DNA vaccine, based on a Semliki Forest virus replicon carrying the *σC* coding gene, was shown to induce specific, neutralizing antibodies, and a marked cellular immune response [79]. Ducklings immunized with this DNA vaccine were completely protected from challenge, showing no clinical signs, mortality, or pathological lesions, and the immunity that developed effectively cleared the virus from the animals.

Another promising approach is the use of live attenuated vaccines. A naturally attenuated novel WRV strain, N20, was found to be safe in ducklings while providing 100% protection against challenge with a virulent strain, significantly reducing viral loads in all tissues [80].

A powerful strategy to overcome the challenge of viral diversity involves creating multivalent vaccines. A bivalent inactivated vaccine, containing two distinct NDRV strains (DE13 and WL01), was developed and provided 100% protection against challenge from both strains [74]. This approach directly addresses the issue of poor cross-protection seen with monovalent vaccines. Furthermore, this bivalent vaccine has been proved to provide long duration of immunity. When administered to pre-laying ducks, the offspring through the egg-yolk received high levels of maternal antibodies, providing protective passive immunity for the initial 21 days of life. Vaccination of breeders has the potential benefit to disrupt vertical transmission of the virus from breeders to ducklings and preventing early infection of ducklings [74].

It is important to note that currently there are no published data on the results of mass vaccination in duck and goose populations, so the reliability and effectiveness of the above experimental approaches under field conditions await formal demonstration.

## 7. Conclusions and Future Directions

Waterfowl reovirus (WRV) infections, particularly those caused by novel WRV strains, continue to be a significant threat, responsible for high-mortality outbreaks, especially in Asia. This has led to a research landscape heavily concentrated in China, leaving a considerable knowledge gap regarding the prevalence, genetic diversity, and economic impact of WRVs in other major waterfowl-producing regions.

The lack of comprehensive viral genetic data from a wider geographical area severely hampers our understanding of global viral dispersal patterns and evolution. Although surveillance reports are scattered, evidence suggests that wild Anseriformes may act as reservoirs for WRVs, potentially introducing new strains to domestic flocks. The intensive nature of modern waterfowl farming, often with shared water sources, likely facilitates transmission and creates opportunities for genetic interaction between circulating viruses. To address these gaps, a future priority must be the implementation of structured molecular surveillance programs. Leveraging the advanced diagnostic tools detailed in this review—from highly sensitive qPCR assays to unbiased viral metagenomics—will be essential for tracking viral spread, identifying novel variants, and understanding the epizootiology and ecology of WRV infections.

Furthermore, a deeper understanding of WRV pathogenesis is needed. The severity of clinical outcomes is complex, influenced by a combination of host factors (age, genetics, immune status), viral factors (strain, genetic constellation, infectious dose), and environmental stressors. The role of bacterial co-infections remains ambiguous; while some studies suggest coinfection with pathogens like *Salmonella* spp. can worsen disease, the presence of others, such as *Escherichia coli* and *Riemerella anatipestifer*, does not consistently lead to more severe pathology [81,82,83]. Future research should aim to untangle these interactions to develop more effective therapeutic and preventative strategies.

Vaccination remains the most critical tool for controlling WRV-associated diseases. Currently, control often relies on autogenous inactivated vaccines administered to breeder flocks. This strategy aims to prevent vertical transmission and provide offspring with durable maternal immunity. However, the primary challenge to developing a broadly protective commercial vaccine is the marked genetic heterogeneity and the unknown antigenic diversity among circulating WRV strains. This diversity explains why traditional monovalent inactivated vaccines often fail, providing excellent protection against homologous viruses but very poor cross-protection against heterologous strains. Fortunately, the research pipeline for next-generation vaccines is robust and the modern approaches offer the potential for safer, more targeted, and more potent immune responses. The crucial next steps will be to establish the full spectrum of circulating serotypes and to advance these promising vaccine candidates through large-scale field trials to confirm their efficacy and safety under real-world conditions, paving the way for their commercial availability.

## Figures and Tables

**Table 1:** Advanced diagnostic methods developed for diagnosis and epidemiological surveillance over the past decade.

Study	Methodology	Target Virus(es)	Target WRV Gene	Sensitivity and Specificity
Zhang et al., 2015 [66]	GeXP analyzer-based multiplex reverse-transcription PCR assay	AIV, DHV, DTMUV, EDSV, DEV, NDV, DuCV, MDRV, MDPV	*σA* gene	10^3^ copies (when all 12 plasmids were present) Specific, no amplification products from EC, S, SA, PM, IBV, MG
Wang et al., 2017 [62]	Multiplex PCR	DHAV-1, DPV, DTMUV, MDPV, MDRV, AIV	*σC* gene	10 copies/µL (single template), 100 copies/µL (combined template) Specific, no cross-reaction with other viruses
Li et al., 2018 [70]	Visual reverse transcription loop-mediated isothermal amplification (RT-LAMP)	NDRV	*σB* gene	200 fg virus-positive RNA extract Specific, no cross-reaction with MDRV, ARV, DPV, DHAV, NDV, AIV, DTMUV
Zheng et al., 2020 [65]	TaqMan-MGB real-time RT-PCR assay with an internal amplification control	MDRV	*σB* gene	2.83 × 10^1^ copies/µL Specific, no cross-reaction with DHAV-1, DPV, DTMUV, MDPV, APMV-1, ARV, NDRV
Zhang et al., 2020 [64]	TaqMan-based real-time PCR assay	NDRV	*σA* gene	10 copies/µL (Ct values: 38.3) Specific, no cross-reaction with AIV, DTMUV, GPV, NGPV, DHAV-1, DHAV-3, DuCV, DPV
Wang et al., 2021 [71]	Reverse transcription recombinase polymerase amplification (RT-RPA)	NDRV	*σB* gene	4.14 × 10^2^ copies/µL Specific, no cross-reaction with MDRV, ARV, DHAV, NDV, DTMUV, DPV, IBDV, IBV, aMPV
Yin et al., 2023 [63]	Multiplex digital PCR	DTMUV, DuCV, NDRV	*σB* gene	1.3 copies/µL Specific, no cross-reaction with MDRV, MDPV, GPV, AIV, NDV
Xu et al., 2024 [69]	RT-qPCR assay with high-resolution melting analysis	C-MDRV, Go-MDRV	*σNS* gene (C-MDRV), *λA* gene (Go-MDRV)	C-MDRV: 5.14 × 10^1^ copies/µL, Go-MDRV: 6.18 × 10^1^ copies/µL Specific, no cross-reaction with GPV, MDPV, DAdV, DTMUV, DEV, NDRV, DHAV-1, DPMV
Wang et al., 2024 [68]	One-Step Multiplex Real-Time Fluorescent Quantitative Reverse Transcription PCR	DTMUV, DHV, MDRV, MDPV	*σB* gene	2.68 × 10^1^ copies/µL Specific, no cross-reaction with FADV, IBDV, IBV, ILTV, HPg, DuCV, GoAstV, MG
Jiang et al., 2025 [72]	RAA-based CRISPR-Cas13a molecular detection system	NDRV	*σC* gene	1 copy/µL Specific, no cross-reaction with DHAV, TMUV, NGPV
Yan et al., 2025 [67]	Visual Gene Chip Method	GPV, DEV, MDPV, DHAV-1, DHAV-3, DTMUV, NDRV	*σC* gene	1 copy/µL for single and mixed samples Specific, no cross-reaction with other pathogens

Abbreviations of pathogen names used in the table: Avian Influenza Virus (AIV), Avian Metapneumovirus (aMPV), Avian Reovirus (ARV), Duck Aviadenovirus (DAdV), Duck Circovirus (DuCV), Duck Enteritis Virus/Duck Plague Virus (DEV/DPV), Duck *Escherichia coli* (EC), Duck Hepatitis A Virus (DHAV/DHV), Duck Paramyxovirus (DPMV), Duck *Salmonella* (S), Duck *Staphylococcus aureus* (SA), Duck Tembusu Virus (DTMUV/TMUV), Egg Drop Syndrome Virus (EDSV), Fowl Adenovirus (FADV), Goose Astrovirus (GoAstv), Goose Parvovirus (GPV), *Haemophilus paragallinarum* (HPg), Infectious Bronchitis Virus (IBV), Infectious Bursal Disease Virus (IBDV), Infectious Laryngotracheitis Virus (ILTV), Muscovy Duck Parvovirus (MDPV), Muscovy Duck Reovirus (MDRV; C-, classical, Go-, goose), *Mycoplasma gallisepticum* (MG), Newcastle Disease Virus/Avian Paramyxovirus type 1 (NDV/APMV-1), Novel Duck-origin Goose Parvovirus (NGPV), Novel Duck Reovirus (NDRV), *Pasteurella multocida* (PM).

**Table 2:** Vaccine candidates developed to prevent WRV infection in waterfowls.

Study	Vaccine Candidate	Vaccine Type/Route of Administration	Vaccine Antigen (Dosage)	Immune Response	Clinical Protection/ Lesions/Virus Load/Virus Shedding
Wang et al., 2025 [77]	rBac-σB	Subunit (Baculovirus-expressed) IM	σB protein (160 ng, single dose)	Induced σB antibodies	No significant weight loss after challenge for all four vaccines. Reduced gross lesions, milder or no histological lesions for recombinant vaccines; somewhat more severe lesions for inactivated vaccine. Reduced viral replication and shedding for recombinant vaccines partly for inactivated vaccine
	rBac-σC	Subunit (Baculovirus-expressed) IM	σC protein (160 ng, single dose)	Induced stronger σC antibody response and higher VN titer than inactivated vaccine	
	rBac-σB+σC	Subunit (Baculovirus-expressed) IM	σB+σC proteins (160 ng each, single dose)	Better protective efficacies than single subunit formulas and inactivated	
	Inactivated NDRV	Whole virus inactivated IM	NDRV WYC strain (5 × 10^−4.5^ EID_50_)	Showed lower σC and VN antibody levels compared to subunit vaccines	
Chen et al., 2024 [78]	Recombinant MLBE of σB protein	Subunit (*E. coli* expressed)	Multiple linear B cell epitopes (MLBEs) of σB protein (10, 20, and 40 µg)	100% serum conversion rate when using higher protein amount (20 and 40 µg)	100% protection, no clinical signs or gross/histopathological lesions, reduced viral load and viral shedding for 20 and 40 µg doses
Huang et al., 2025 [74]	Inactivated Monovalent DE13	Whole virus inactivated SC	NDRV DE13 strain (10^8^ TCID_50_/mL)	For monovalent vaccines, virus neutralizing titer against homologous virus significantly higher than heterologous For bivalent vaccine, rapid immunity (within 2 weeks), high neutralizing antibodies in breeders, persisting for 80 days in serum and yolk	For monovalent vaccines, 100% clinical protection against homologous virus challenge, partial (13.3% to 26.7%) protection against heterologous virus For bivalent vaccine, 100% protection against both challenge viruses. Passive immunity in offspring provides 100% protection until 21 days of age.
	Inactivated Monovalent WL01	Whole virus inactivated SC	NDRV WL01 strain (10^8^ TCID_50_/mL)		
	Inactivated Bivalent (DE13 + WL01)	Whole virus inactivated SC	NDRV DE13 + WL01 strains (10^8^ TCID_50_/mL of each strain)		
Yan et al., 2021 [80]	Naturally Attenuated N20 strain	Live attenuated vaccine IM	N20 strain (100 ELD_50_/mL)	Generated NDRV-specific antibodies at D6. Sera neutralized different NDRVs.	100% protection against virulent N19 challenge. No clinical signs, gross/histopathological lesions, or body weight loss. Reduced viral replication and shedding
Bi et al., 2016 [76]	Recombinant σC protein (TH11)	Subunit (Baculovirus-expressed) SC	Purified σC protein (5 µg σC protein, two doses, 2-week intervals)	Robust humoral and cellular immune responses. High neutralizing antibodies. Elevated IFN-γ and IL-4 levels	100% protection against lethal challenge. Mild signs of illness. Reduced viremia, decreased virus replication
Zhu et al., 2015 [79]	Suicidal DNA vaccine of *σC* gene	DNA vaccine (SFV replicon based) IM	*σC* gene of NDRV (100 µg as primary, 200 µg as booster)	Induced NDRV-specific antibodies, neutralizing antibodies, IFN-γ and IL-4.	100% protection against challenge. No clinical signs or mortality. No lesions. Undetectable levels of virus by RT-PCR. Reduced viremia, decreased virus replication
Niu et al., 2017 [73]	Inactivated GRV Vaccine	Whole virus inactivated IM	GRV JS-01 strain (0.2 mL or 0.5 mL allantoic fluid)	Antibodies detectable at 6 days post-vaccination, peaked at 3 weeks, ELISA values of serum antibody correlates with protection	At 12 dpi with 0.5 mL vaccine, 100% protection observed. Reduced or no lesions. Lower viral shedding in vaccinated groups.
Kuntz-Simon et al., 2002 [75]	Baculovirus-expressed σC	Subunit (Baculovirus-expressed) SC	σC protein (DRV-89330 strain) (1 × 10^7^ infected cell equivalents (1st dose), 1.5 × 10^7^ (2nd dose)	Elicited DRV-specific neutralizing antibodies. Antibodies detected in 100% of sera	For both vaccines, partial or full prevention of clinical signs and reduced severity of tenosynovitis For the bivalent vaccine, less lesions than σC alone.
	Baculovirus-expressed σC+σB	Subunit (Baculovirus-expressed) SC	σC + σB proteins (DRV-89330 strain) (1 × 10^7^ infected cell equivalents (1st dose), 1.5 × 10^7^ (2nd dose)		

## Data Availability

The sequence alignment used for the phylogenetic tree (Figure 2) is available from the corresponding author upon request.
